# 3D-printable zwitterionic nano-composite hydrogel system for biomedical applications

**DOI:** 10.1177/2041731420967294

**Published:** 2020-10-29

**Authors:** Nathalie Sällström, Andrew Capel, Mark P Lewis, Daniel S Engstrøm, Simon Martin

**Affiliations:** 1 Wolfson School of Mechanical Electrical & Manufacturing Engineering, Loughborough University, Loughborough, Leicestershire, UK; 2 School of Sport, Exercise and Health Sciences, Loughborough University, Loughborough, Leicestershire, UK; 3 Department of Materials, Loughborough University, Loughborough, Leicestershire, UK

**Keywords:** Nanocomposite, zwitterionic hydrogel, printing-then-curing approach, sulfobetaine methacrylate

## Abstract

Herein, the cytotoxicity of a novel zwitterionic sulfobetaine hydrogel system with a nano-clay crosslinker has been investigated. We demonstrate that careful selection of the composition of the system (monomer to Laponite content) allows the material to be formed into controlled shapes using an extrusion based additive manufacturing technique with the ability to tune the mechanical properties of the product. Moreover, the printed structures can support their own weight without requiring curing during printing which enables the use of a printing-then-curing approach. Cell culture experiments were conducted to evaluate the neural cytotoxicity of the developed hydrogel system. Cytotoxicity evaluations were conducted on three different conditions; a control condition, an indirect condition (where the culture medium used had been in contact with the hydrogel to investigate leaching) and a direct condition (cells growing directly on the hydrogel). The result showed no significant difference in cell viability between the different conditions and cells were also found to be growing on the hydrogel surface with extended neurites present.

## Introduction

Hydrogels are insoluble polymeric networks which have the ability to absorb large amounts of water (from ~10% to ~1000 times of their dry weight)^1^ and have been widely studied for various different biomedical applications, including wound healing, drug delivery and tissue engineering. Hydrogels have shown numerous tissue engineering applications since they, to some extent, mimic the extracellular matrix of tissues, attributable to their high water content and porous structure.^2^ However, their usage is somewhat limited due to their poor mechanical strength, brittle nature and low fracture toughness.^3^ To improve the mechanical properties of hydrogels, different methods have been used, including; adding functional end groups, using sliding crosslinks (crosslinks which can move when stress is applied to prevent stress concentration),^4^ though incorporating nano-structures (nano-composites) or microparticles and also by using a secondary network.^5^ Herein, nano-composites will be used to reinforce the hydrogel system. Nano-composites can naturally exist within biological systems as hybrids of organic and inorganic compounds such as in teeth and bones.^6^ One type of nano-composite which has been previously used in hydrogels is Laponite. Laponite is a synthetic hectorite nanoclay ([Mg_5.34_Li_0_._66_Si_8_O_20_(OH)_4_]Na_0.66_) which has previously been used as a crosslinker within different hydrogel systems including; Poly(ethylene glycol) diacrylate (PEGDA),^7^ N-isopropylacrylamide (NIPA),^8^ N,N-dimethylacrylamide (DMAA)^9^ and zwitterionic sulfobetaine monomers.^10,11^

Laponite consists of discs (1 nm thick and 25 nm in diameter) that are made up from tetra-octa-tetra (TOT) layers. The O layer consist of a Mg-O octahedral sheet which is sandwiched between two T layers consisting of Si-O tetrahedral sheets. Net negative charges can occur on the surface through isomorphous substitution by substituting Mg^2+^ with Li^+^ in the octahedral sheet. Exchangeable cations like Na^+^ can therefore exist within the layers. Negative charges also occur as Laponite is dispersed in water, since the Na^+^ Ions will dissociate from the individual clay platelets. Positive charges occur around the edges of the clay sheets due to OH^-^ ions dissociating from the edges of the clay platelet.^12^ It is this charge distribution which leads to the clay forming what is known as a ‘house-of-cards’ structure in aqueous suspensions, due to ionic interactions. When Laponite is dispersed in water, functional groups present on the surface will be exposed which will allow attachment of several polymer chains to one clay platelet (for chemical crosslinkers this is not the case).^3^ It is this that contributes to the increased mechanical properties seen for nano-composites.

Laponite suspensions also show interesting rheological properties. They possess thixotropic behaviours (gradual increase in viscosity over time) due to the formation of the ‘house-of-card’ structure^13^ as well as shear thinning behaviours. This is therefore also a material of interest when it comes to additive manufacturing (AM). Hydrogels containing Laponite clay as a crosslinker has previously been printed successfully using extrusion based AM methods,^12^ however, no zwitterionic nano-composite hydrogel has previously been produced though AM. AM is of interest in tissue engineering since it is possible to achieve highly controlled macro and micro structures, printing scaffolds, as well as the possibility of incorporating and spatially controlling cells and growth factors into the structures during the printing process.^14,15^ A range of applications has been proposed for additively manufactured hydrogels including aortic valve hydrogel scaffolds,^16^ neural guidance scaffolds^17^ and for the fabrication of human skin.^18^ For biomaterials, it is also important to be able to control the cellular behaviours on the material surface. To do this, there are three main cues that need to be considered; chemical, mechanical and topological. One way of controlling cell growth is to use polymers which are either non-fouling to cells or cell adhesive. Non-fouling polymers usually have either a strong surface hydration (such as PEG) or very low surface energy.^19^ Zwitterionic polymers also display non-fouling properties which may be due to the balanced charged of the zwitterions preventing protein adsorption from occurring.^20^ Herein, the aim is to develop a non-fouling hydrogel to use in combination with a cell adhesive coating in order to selectively grow and direct neurons.

In this work, Laponite clay is used as a crosslinker for a zwitterionic monomer (N-(3-Sulfopropyl)-N-methacroyloxyethyl- N,N-dimethylammonium betaine (SPE)) to create a mechanically robust hydrogel system which can be produced through an extrusion-based AM technique. We hypothesised that the SPE monomer would be non-fouling since it has a similar structure to KSPMA (3-sulfopropyl methacrylate potassium salt) which has previously shown non-fouling characteristic and has been used in combination with a 2-bromoisobutyl bromide (BIBB) functionalised surface to selectively grow neurons on the BIBB surface but not the KSPMA surface.^21^ The KSPMA monomer could not be used within the nanoclay hydrogel system due to charge interactions between the monomer and the Laponite platelets, preventing the clay from working as a crosslinker which is why SPE is evaluated herein. Current non-fouling hydrogels are commonly developed using PEG, however one issue with these hydrogels are that PEG has shown oxidative degradation. This means that these hydrogels may not be suitable for long-term implantation in a biological environment due to the presence of oxidisers which could lead to the material losing its non-fouling properties.^22^ Another type of non-fouling monomer which has been shown to be a sturdy alternative to PEG is zwitterionic monomers, such as sulfobetaine.^23^ Current sulfobetaine monomers used within nano-clay systems are cured using thermal methods^10,11^ which can be somewhat challenging when attempting to print the material through AM. The material developed herein will therefore be cured though UV-photopolymerisation which will allow more control of the curing process.

The application in mind while developing this material is additively manufacturable fully integrated socket components for next generation lower limb prostheses. Current prosthetic sockets are typically made from hard polymeric materials such as polypropylene based materials or through the use of laminate composites.^24^ Furthermore, residual limbs are prone to dimensional changes over time, particularly in the first 12 to 18 months after amputation due to muscle atrophy, however, this also continues after 18 months.^25^ The current socket materials do not adapt to these dimensional changes which lead to poorly fitting and uncomfortable sockets. The material developed herein aims to be soft to adapt to these changes in dimensions and at the same time be biocompatible within the neural environment to establish a neural connection between the user and the device for improved control and feedback. The aim is for this soft material to transfer the load from the residual limb onto a structural outer layer. This means that the developed material does not need to have the same load bearing capabilities as the current sockets. To achieve a neural connection, the aim is to achieve a non-fouling hydrogel surface to use in combination with a cell adhesive coating to selectively grow neurons towards electrode surfaces which can transmit the neural signal.

Mechanical forces present within a lower limb prosthetic socket includes compression, shear and tension.^26^ Tension and shear forces occur during the swing though phase of the gait while compression forces occur during the stance phase of walking. These properties therefore need to be considered when developing a socket material. As the socket is aimed to be produced though AM, the tensile properties will be evaluated herein as this will allow evaluation of the bonding strength between printed strands which can be compared to cast samples. Evaluation of the compression properties of this hydrogel system has previously been published.^27^ Rheological evaluations were performed to evaluate the printability of different compositions and the cytotoxicity of the system was evaluated though culture of neuronal cells.

## Materials and methods

### Hydrogel preparation

Laponite XLG (BYK Additives) was first dispersed in nitrogen purged deionised (DI) water by magnetic stirring for 20 min. The monomer, N-(3-Sulfopropyl)-N-methacroyloxyethyl- N,N-dimethylammonium betaine (SPE) (Merck Chemicals, UK, used as received) were then mixed in the dispersed Laponite at a concentration of either 10wt% SPE 6wt% Laponite, 50wt% SPE 6wt% Laponite or 10wt% SPE 10wt% Laponite. Previous experiments in the lab showed that Laponite contents below 6wt% (for 10wt% SPE) resulted in the material being unable to hold its shape and could thus not be printed using extrusion based additive manufacturing. The 10wt%SPE 10wt% Laponite composition was too viscous to mix using magnetic stirring and had to be mixed manually. Once fully dispersed a photoinitiator, Irgacure 2959 (Sigma-Aldrich, UK), was added at 0.4wt%. For cast samples, the suspension was directly poured into tensile sample moulds (ASTM D638-14 type IV dimensions, produced though AM using PLA filaments in an Ultimaker 2 printer). These were then UV-cured at 365 nm for 1 h at an intensity of 2200 μW/cm^2^.

### Rheological evaluation

Rheological measurements of the pre-gel suspensions were performed at room temperature using a HAAKE viscometer IQ (Thermo Fisher) with a concentric cylinder geometry. A shear ramp test was used, starting from 0.01s^-1^ and going up to 100s^-1^ during 120 s. 15 s of pre-shear of 0.01s^-1^ was used before the shear ramp started. This test was conducted on all compositions and was tested both directly after the pre-hydrogel solution was prepared and 24 h after preparation.

### Additive manufacturing

**Figure 1.**
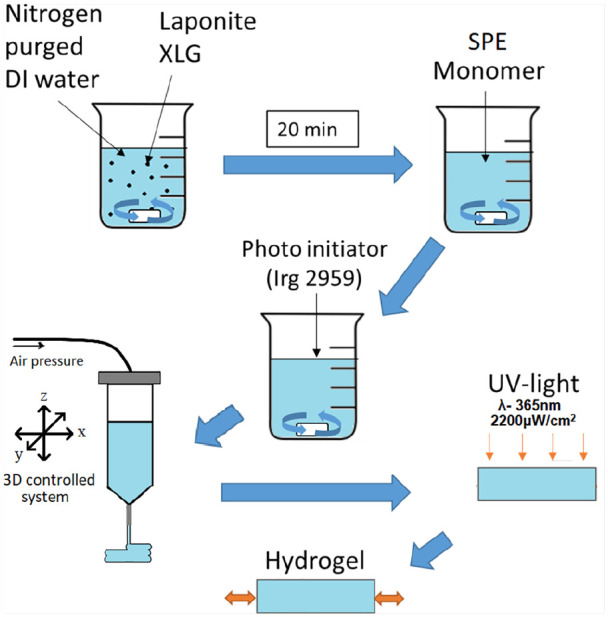
Schematic diagram of the hydrogel preparation and manufacturing method.

Additive manufacturing of the pre-gel suspension was conducted using a syringe based dispensing technique (Bioplotter, EnvisionTEC). Directly after preparation, the pre-gel was loaded into a syringe and left to settle for 24 h prior to printing. A diagram of the process can be observed in Figure 1. A dispersing needle with an inner diameter of 1 mm was used for printing. Line printing was used as a tool to evaluate the most suitable printing parameters to use for this material. The parameters with the linewidth most similar to the nozzle diameter (1 mm) was then chosen (this was 0.8 bar at a speed of 10 mm/s). Tensile samples were printed using two different dispersion orientations, longitudinal (toolpath along the testing direction) and transverse (toolpath perpendicular to the testing direction). Printing was conducted at room temperature, after printing, the material was UV-cured at λ-365 nm for 1 h at an intensity of 2200 μW/cm^2^ with a distance of 20 mm between the samples and the UV source.

### Mechanical testing

Tensile samples were printed using the previously described method using dimensions of the ASTM D638-14 type IV standard with all dimensions scaled by a factor of 0.5 to fit the printing platform. Cast samples were moulded directly after the pre-gel preparation using moulds produced though AM (ASTM D638-14 type IV). Uniaxial tensile testing was performed using a universal testing machine (Instron 5944) fitted with a 2 kN load cell. All of the printed and cast samples were tested at a strain rate of 10 mm/min, the software (Bluehill 3) automatically calculated the stress, strain and Young’s modulus of the samples. Eight samples were tested for each condition.

### Swelling evaluation

Swelling of cast hydrogels were conducted in both water and cell culture medium (DMEM + Glutamax with 1% penicillin-streptomycin) in an incubator at 37°C with 5% CO_2_ for up to 7 days. Samples were weighed before swelling and after swelling for 1, 3 and 7 days (*n* = 7). The swelling percentage (Q) was calculated using Q = ((W_S_-W_o_)/W_o_) × 100, where W_S_ is the weight of the swollen hydrogel at a particular time and W_o_ is the weight of the original hydrogel.

### Cell culture

The SH-SY5Y neuroblastoma cell line (ECACC) was cultured in T80 flasks (Nunc™, Fisher Scientific, UK) in growth media (GM), composed of 89% Gibco™ Dulbecco’s Modified Eagles Medium (DMEM) supplemented with GlutaMAX (Fisher), 10% fetal bovine serum (FBS, Pan Biotech, UK), 1% penicillin-streptomycin (Fisher) until 80% confluence was attained, prior to being harvested for experimental use. SH-SY5Y cells were cultured for 48 h in GM prior to inducing neuronal differentiation via medium (DM) containing GM supplemented with 1μM retinoic acid (Sigma) differentiating agent for a further 72 h. All cell lines were incubated within a 5% CO_2_ atmosphere at 37°C (HERAcell 240i, Thermo Fisher, UK) for the duration of all experiments.

### Experimental treatments

Experiments were designed to simultaneously assess critical evidence of cellular viability and morphological observations, in response to the direct (cells cultured directly on the hydrogel) and indirect (cells cultured within chemical leachate) effects of the hydrogel (10SPE 6Laponite composition). After preparation and moulding, the hydrogels were swollen in DI water for 72 h to reach swelling equilibrium (to prevent changes in dimension during cell culture). The gels were cut to a diameter of approx. 22 mm to fit within the confines of a 12 well-plate (Nunc™, Fisher). The hydrogels were sterilised using 70% ethanol, washed in sterile phosphate buffered saline solution (PBS), and finally irradiated under UV light for 3 h. The hydrogels were then immersed (pre-treated) in un-supplemented DMEM for 48 h with a daily change of medium, followed by a further 24 h in GM. This GM was saved for experimental treatments and a selection of these wells remained aceullar and continued to be replenished with either fresh GM or DM daily (MEDIA ONLY). Samples were used to evaluate the direct (*n* = 3) and indirect (*n* = 3) biocompatibility of the hydrogel per repeat (*n* = 3), totalling *n* = 9 analysis points per condition. To assess direct biocompatibility 5×10^4^ SH-SY5Y cells were seeded either directly (DIRECT) onto the hydrogels or into control (CON) wells along with 2 mL of fresh GM. To assess the indirect biocompatibility of the hydrogel leachate, cells were seeded directly into 12 well plates along with 2 mL of hydrogel leachate GM from the MEDIA ONLY wells. The cells within the indirect condition continued throughout the experimental timeline to be supplemented with media (GM or DM) leached from these wells. Cells were analysed for viability at 48 h (End GM) and 120 h (End DM) and fixed for fluorescence imaging.

### Fluorescence staining and microscopy

Cells were washed twice in 2 mL PBS per well, fixed in formaldehyde solution (Sigma, 3.7%) and permeabilised (Triton X-100, Fisher, 1:500). Cells were blocked in 5% goat serum (Fisher) solution for 30 min, and labelled with monoclonal mouse anti-β-tubulin III (1:200; Abcam, UK) antibody for 24 h, prior to being counterstained with Alexafluor 488-conjugated goat anti-mouse IgG (1:200, Life Technologies, Fisher) secondary fluorochrome for 1 h. DAPI nucleur stain (1:2000, Life Technologies, Molecular Probes) was used to stain nuclei. Alexafluor 488 appears green and is indicative of tubulin expression at standardised exposures. DAPI was excited at 358 nm and emitted at 461 nm and appears blue. Light microscopy images were captured on a Leica DMIL LED microscope. Fluorescence images were visualised using a Leica DM2500 microscope with manufacturers software (Leica application suite). Images were analysed using IMAGE J 1.50a/Fiji (Java 1.6.0_24) software. Image inclusion criteria were set at ⩾5 images per well.

### Cell viability alamarBlue^®^ assay

Cellular viability reagent, alamarBlue^®^ diluted 1:10 in GlutaMAX supplemented DMEM was used to assess cell viability and proliferation at 48 and 120 h experimental time-points. Cells were washed twice with 2 mL PBS prior to being treated with 2mL per well alamarBlue^®^ stock solution and humidified 5% CO_2_ at 37°C for 4 h. 100 μL per well of solution was then added to a black well 96-well plate and analysed for fluorescence intensity. Increased fluorescence of alamarBlue^®^ reagent is indicative of an increase in cellular viability. alamarBlue® fluorescence signal was excited at 540 to 570 nm (peak excitation: 570 nm) and emitted at 580 to 610 nm (peak emission: 585 nm).

### Statistical analysis

Statistical analyses and significance of data were determined using IBM^©^ SPSS^©^ Statistics version 22. Mauchly’s test of sphericity and Shapiro-Wilk tests were used to confirm homogeneity of variance and normal distribution of data respectively. Where parametric assumptions were met, a 2 × 2 ANOVA was used for alamarBlue^®^ analyses. One-way ANOVA was used to analyse morphological data only concerned with DM120 h time-point. Where significant interactions were observed, independent t-tests (*t*) were used to analyse differences between conditions at specific time-points. All data is reported as mean ± standard deviation (SD). Significance was assumed at *p* ⩽ 0.05.

## Result and discussion

### Rheological evaluation

**Figure 2.**
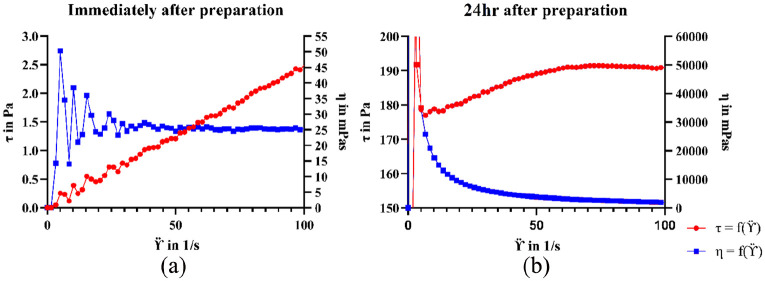
Viscosity of the 10SPE 6Laponite pre-hydrogel suspensions. (a) Viscosity measured immediately after preparation. (b) Measured 24 h after preparation. The blue curve represents the viscosity (η)in mPas and the red curve the shear stress (τ) in Pa.

The viscosity measurements of different pre-gel suspensions demonstrated that the material displayed time dependent viscosity (thixotropic properties). Directly after preparation, the pre-hydrogel suspensions were found to have low viscosities (below 25 mPa-s) with Newtonian behaviour (Figure 2). After 24 h, the viscosity increased, and the suspensions displayed shear thinning behaviours. For extrusion-based printing, shear thinning, and thixotropic properties are important for successfully extruding the material though the nozzle and for the material to regain its rheological properties to support its own weight. The thixotropic and shear thinning properties of nanoclay hydrogels has previously been shown to be attributed to the disrupting and reformation of the clays ‘house-of-cards’ structure.^12^ As the pre-gel suspension do not show shear thinning behaviours immediately after preparation, these are not suitable for extrusion-based printing and thus requires aging. Due to this, the printing conducted in this work was using material which had been aged for 24 h. The effect on aging on the rheological properties of this material has previously been published.^27^

Furthermore, the rheological properties varied with monomer to Laponite concentrations where an increase in Laponite content resulted in increased viscosity and an increase in the monomer content resulted in reduced viscosity (Table 1). This could be caused by ionic Laponite platelet interactions leading to ‘house-of-cards’ formations. With increased clay content, there is a rise in platelet interactions which require higher forces to disrupt leading to an increase in viscosity. If there is an excess of monomer present in relation to the Laponite clay (as the 50SPE 6Laponite composition), the viscosity does not significantly increase with time, which may indicate that the ‘house-of-cards’ structure cannot be formed. If the Laponite content is the same as the monomer content (10SPE 10Laponite composition) it makes the suspension highly viscous even directly after preparation which disrupts the mixing process. This composition also showed shear thinning behaviours directly after preparation which could indicate that the ‘house-of-cards’ structure is formed earlier due to increased charge interactions.

**Table 1.** **Shear stress and viscosity data for different pre-gel suspensions.** All data points were taken at a shear rate (ϔ in 1^-s^) of 50.

Composition (WT%)	Immediately after preparation		24 hours after preparation	
	τ (Pa)	η (mPa-s)	τ (Pa)	η (mPa-s)
**10SPE 10Laponite**	162.75	4.40	441.59	11.96
**10SPE 6Laponite**	1.86	25.00	191.43	2567.61
**50SPE 6Laponite**	0.28	0.01	8.14	0.22

### Additive manufacturing

**Figure 3.**
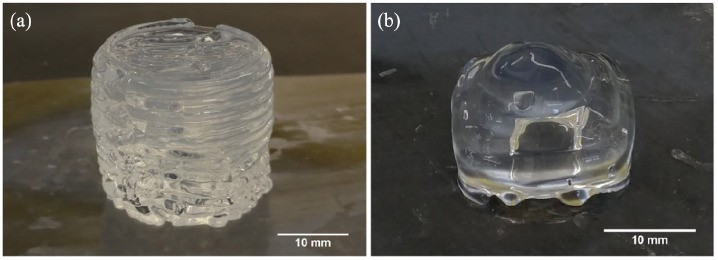
Printed uncured SPE hydrogel structures. (a) Printed cylinder. (b) Printed pyramid with a hollow centre.

The 10SPE 6Laponite composition was used in the printing process based on the result from the viscosity measurements. The 10SPE 6Laponite composition displayed increased viscosity over time (which the 50SPE 6Laponite composition lacked) and did not display difficult mixing during gel preparation (as the 10SPE 6Laponite composition). Structures with simple geometries were successfully printed, as seen in Figure 3(a) and (b), several layers of the material could be deposited without the need of curing in-between the layers. A pyramid shape with a hollow centre was printed to further evaluate the self-supporting abilities of the material. The structures were shown to be able to support themselves without collapsing and this could be maintained during and after curing was completed. This developed hydrogel system therefore appears to be suitable to be used in a printing-then-curing approach which could reduce the problem with intralayer adhesion for printed parts. Nano-clay based hydrogel systems have previously been shown to be self-supporting for additive manufacturing applications in their uncured state.^12,28^ The self-supporting nature is caused by a disruption and recovery of the ‘house-of-cards’ structure. Once sufficient stress is applied to the nano-clay material, the ‘house-of-cards’ structure gets disrupted causing a drop-in viscosity. This was also observed from the viscosity measurements conducted herein (Figure 2).

### Mechanical testing

**Figure 4.**
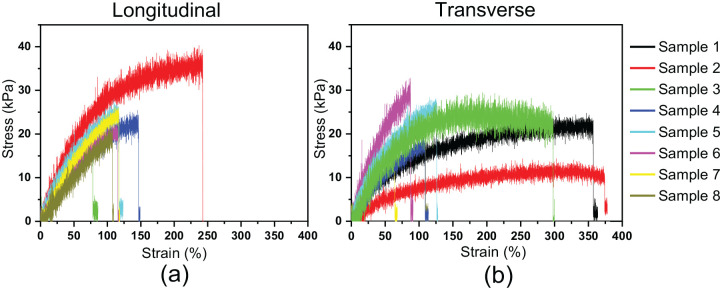
Tensile testing of printed samples after UV-curing. Stress and strain curves for (a) Longitudinal printed samples. (b) Transversely printed samples.

**Figure 5.**
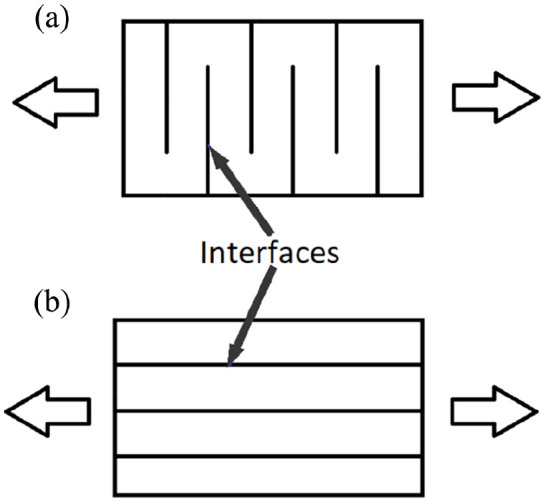
Simplified diagram of the printing directions and the interfaces between the extruded material strands for (a) Transverse printing direction (b) Longitudinal printing direction.

One-layer thick tensile samples were printed to evaluate the effect of different printing direction on the tensile properties. This was conducted to evaluate the bond-strength between the printed strands (transverse direction) compared to samples printed in a longitudinal direction which does not directly test the strength between the interfaces. The printed samples were also compared to cast bulk samples. The stress at failure for both printing directions were around 20 kPa with some sample variation (Figure 4). A larger sample variation was seen for the transversely printed hydrogel, particularly in terms of elongation at break. The cast samples had about twice the stress at failure compared to the printed samples as well as a significantly larger elongation at break (Table 2). It is possible that the large sample variation observed for the transversely printed samples were caused by an increase in interfaces with weaker bonding in-between which may be causing a more uncertain breaking pattern. It is possible that some of the interfaces are breaking but there is a partial interface in the corners (due to the print head changing direction) (Figure 5) which may be stronger than the full interfaces, causing stretching of the corners rather than breaking (thus the increase in strain seen in some cases). In the lower strain samples observed, it may be that the samples broke across the interfaces rather than stretching. The cast samples also displayed a large sample variation for elongation at break (259%). However, when considering the high mean value of the elongation at break (865%) the sample variation is still proportionally significantly lower that the printed samples. The cast samples displayed a variation in elongation of break of 30% while the longitudinal printed samples displayed a variation of 53% and transversely printed samples a variation of 64%.

**Table 2.** Tensile data of hydrogels with different manufacturing methods with the same composition (10SPE 6Laponite). Values are reported as mean ± standard deviation (SD) eight samples for each manufacturing method.

	Stress at break (kPa)	Elongation at break (%)	Youngs modulus (kPa)
**Cast (non-aged)**	40.9 ± 17.0	864.7 ± 258.8	31.7 ± 19.3
**Longitudinal print**	21.0 ± 9.8	116.4 ± 61.7	33.1 ± 8.3
**Transverse print**	20.0 ± 5.5	190.5 ± 120.9	42.5 ± 10.1

Moreover, the greatest influence on tensile properties does not appear to be the printing direction but rather the printing process itself, since the casted samples had a significantly higher failure stresses and strains (Table 2). This could potentially be due to the aging of the pre-gel material, since the material used for printing is left to settle for 24 h before curing, while the casted samples are cured directly after preparation. As seen from the viscosity measurements (Table 1), there is a significant increase in viscosity after leaving the suspension out for 24 h which may have an effect on the polymerisation process and thus reducing the mechanical properties of the hydrogels. The effect of aging on SPE nano-composite hydrogels with the same composition has previously been reported.^27^ The result showed that aging of cast hydrogels for 48 h before curing causes a significant drop in tensile properties (elongation at break: 239.7 ± 51.2% and stress at break: 28.7 ± 6.3 kPa) compared to non-aged cast samples, this reduction in properties could be reversed for the printed samples by printing at a slower speed and curing the material while it is printing.^27^ This result is comparable to the result for the printed samples herein which were aged for 24 h before curing where an elongation of break of 190.5 ± 120.9 % and stress at break of 20.0 ± 5.5 kPa was achieved (transversely printed samples) (Table 2). Herein a curing time of 24 h was used and not 48 h, however, data shows that there is no significant difference in tensile properties of samples aged for 5 to 9 h and 48 h before curing (see Figure S1 and Table S1) indicating that 24 h aging should show similar result to the 48 h aging time.

Zwitterionic sulfobetaine nano-clay composites has previously been developed,^10,11^ however, to the knowledge of the authors, the monomer used herein, SPE, has not been previously used within this type of hydrogel network. The previous sulfobetaine monomers used contained an amine rather than an oxygen molecule which may have some influence the mechanical properties. However, as no zwitterionic nano-clay system have previously been printed, the result in this work can only be compared to cast hydrogel samples of a similar composition. Ning et al. 2013 reported on the mechanical properties of a similar hydrogel made with the monomer 3-dimethyl-[3-(N-methacrylamido)propyl]ammonium propanesulfonate (M_3_) and Laponite clay (NC).^29^ The cast samples developed in this work had a much higher stress at break (40.9 ± 17 kPa) compared to the sulfobetaine clay hydrogels developed by Ning et al. (2.9 kPa for the M_3_−NC_3_ hydrogels which had a clay content of 2.286wt%). This improvement in stress at break could be caused by the higher clay content used in this work (6wt%). The elongation at break recorded herein (864.7 ± 258.8%) is similar to that recorded by Ning et al. (960%).^29^

The conditions present within the soft layer of a fully integrated prosthetic socket is likely similar to the conditions which the soft tissues are exposed to within a prosthetic socket. A study by Portnoy et al. showed the forces present within a prosthetic socket during static conditions, the highest values occurred below the tibia where tensile strains of 129% and stresses of 263 kPa were recorded.^26^ The strains achieved for the material developed herein are similar (116.4 ± 61.7 % for the longitudal printed samples) but significantly lower stresses are achieved (~20kPa). However, the same research group did further studies which showed that the forces present within the socket is patient dependent as well as dependent on the fit of the socket.^30^ In some cases tensile stresses of only 5 kPa was achieved, likely caused by a well-fitting socket which could evenly distribute the force throughout the socket.^31^ The tensile forces achieved herein may therefore be sufficient however further studies would be required to confirm this.

### Swelling evaluation

**Figure 6.**
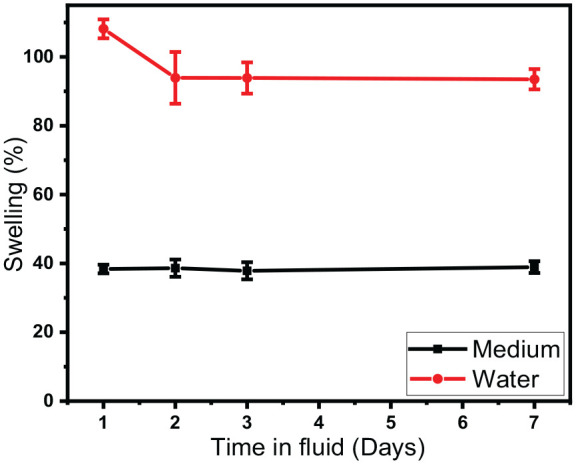
Swelling study of cast hydrogels (10SPE 6Laponite composition) in water and culture medium over 7 days at 37°C.

Hydrogels were swollen to evaluate when swelling equilibrium occurs (Figure 6). The result showed that maximum swelling occurs during the first day and for the hydrogel swollen in water, some de-swelling occurred from day 1 to day 2. This de-swelling is likely due to the Na^+^ ions present within the hydrogel structure (from the Laponite platelets) which diffuses out of the hydrogel into the surrounding water and thus affecting the osmotic pressure causing the hydrogel to de-swell. This swelling-deswelling characteristics of nanoclay hydrogels has previously been explored in literature.^32,33^ Ren et al. utilised inductively coupled plasma atomic emission spectroscopy on the water used to swell the hydrogels in and demonstrated that there are Na^+^ ions present in the water. The Na^+^ ions were mostly released in the early stages of swelling and by the time equilibrium swelling was achieved, 99% of the Na^+^ ions were released indicating that the deswelling is caused by release of Na^+^ ions.^32^ There is also a significant difference in swelling between the different liquids, with less swelling occurring in culture medium (~40%) compared to the water (~100%). This is likely caused by the increased solutes present in the culture medium which results in less water being transported into the hydrogels (due to osmosis). Previous studies on nanoclay hydrogels has shown that hydrogel swelling is reduced with increased ionic strength (both when swelling in NaCl and CaCl_2_ solutions) due to a reduction in osmotic pressure.^34^ Furthermore, osmosis is not the only factor affecting hydrogel swelling, there is also an elastic force present within the hydrogel system which prevents the hydrogel from deforming. When the hydrogel is swollen to equilibrium, the osmosis and elastic forces are balanced and no further swelling may occur.^35^ Previous studies have shown that hydrogel swelling also depends on temperature,^36^ when conducting swelling experiments it is therefore important to do so with the final application in mind, therefore 37°C was used in this case.

### Cytotoxicity evaluations

**Figure 7.**
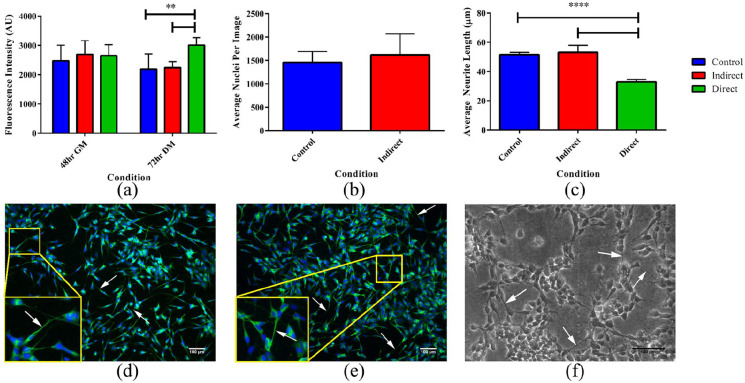
Graphs and images from the cell culture study. (a) AlamarBlue^®^ assay data after 48 h in GM and after 72 h in DM, a significant difference in fluorescence observed between the direct and control condition and between the direct and indirect condition after 72 h in DM. (b) Average nuclei count for the different conditions. (c) Average neurite length for the different conditions measured from the fluorescently stained images and from the phase contrast image (for the direct condition). A significant difference in neurite length observed between the direct and control condition and between the direct and indirect condition. Data presented as mean ± standard deviation from *N* = 3 experimental repeats in each condition. *P ⩽ 0.05, **P ⩽ 0.01, ***P ⩽ 0.001, ****P ⩽ 0.0001. (d) Fluorescently stained SH-SY5Y cells from the control condition (no hydrogel present). (e) From the indirect condition (culture medium previously used for soaking hydrogels). (f) Phase contrast image of the SH-SY5Y cells grown directly on the hydrogel surface. Arrows indicate the location of neurites.

Cells grown on the equilibrium swollen and sterilised hydrogels (10SPE 6Laponite) were shown to survive in culture for at least 5 days. No significant difference in fluorescence were found between the control, direct and indirect samples from the alamarBlue^®^ assay performed after 48 h in GM (Figure 7(a)), however, after 72 h in DM the direct sample displayed a higher fluorescence compared to the indirect and control (Figure 7(a)). After 72 h in DM, the cells were fixed and stained with a nuclei stain (Dapi) and β-tubulin stain (fluorescent antibody) for further evaluation of the cell-hydrogel interaction (Figure 7(d) and (e)). No significant difference in nuclei count between the control and the indirect samples were seen (Figure 7(b)). The direct samples could not be imaged with the stain as the hydrogel absorbed the fluorescent dye as well as the cells, therefore only phase contrast images of the cells growing on the hydrogels could be obtained (Figure 7(f)). The average neurite length was also calculated from the fluorescent and phase contrast images for the different conditions (Figure 7(c)). No significant difference was seen between the control and indirect conditions but a reduced neurite length for the direct condition was observed. This may be due to various reasons which cannot be determined without further studies. It is possible that subtle alterations in the topography or modulus of the hydrogel supported cellular proliferation but limited neurite differentiation when compared to a gold-standard control (Nunc™ cell culture plastic). Further studies would be required to optimize the hydrogel to improve neurite extension.

The alamarBlue^®^ cytotoxicity assay showed no significant reduction in cell viability between the control, direct and indirect samples, indicating that the material developed in this work is not cytotoxic to the SH-SY5Y neuroblastoma cell line, making it potentially suitable for being used in neural applications. It is worth noting that we have not yet attempted to optimise the elastic modulus of the hydrogel system for the growth of the cell systems. An optimisation of the modulus could potentially further improve the neural viability as well as increasing neurite extension. There was also no significant difference in average nuclei count between the indirect and the control, confirming the non-toxic nature of the material. No automated cell count could be made of the direct sample, since the hydrogel also absorbed the fluorescent dye. Consequently, only phase contrast images of the direct sample could be obtained (Figure 7(f)). This meant that it was not possible to evaluate if cells were growing within the hydrogel structure and not only on the surface. To overcome this problem, techniques such as optical projection tomography has previously been used.^37^ The phase contrast images did confirm that there were viable cells present on the hydrogel surface, allowing measurement of the neurite extensions to be undertaken. The cells did appear to preferentially grow in clusters on the surface of the hydrogel, indicating some requirement to optimise the material properties for specific biomaterials applications. To selectively grow cells on the surface, a cell adhesive coating could be used. As mentioned previously, the SPE monomer was hypothesised to possibly have non-fouling properties since it has a similar structure to KSPMA monomer, which has previously been shown to have non-fouling properties^20^ and Zwitterionic monomers are also known for their non-fouling abilities. However, SPE contains an amide group, which is well known to improve cell adhesion.^38^ In addition to this, Laponite clay has been shown previously to aid in cell adhesion of non-fouling materials^39,40^ which may be why cells were able to attach and grow on the hydrogels in this work. This type of hydrogel system has not been tested in cell culture before, however, Huang et al. used a similar sulfobetaine monomer with nano-clay and poly(ethylene glycol) dimethacrylate as a crosslinker. They demonstrated no negative effect on cell viability, in fact the nano-clay containing gels showed increased viability.^11^

The result obtained in this work indicates that the hydrogel system developed is potentially useful for neuronal scaffold applications. Both because the use of extrusion based AM techniques has previously shown to provide a physical cue for axonal guidance^17^ and the material showed no indication of being cytotoxic to the neuroblastoma cell line used. The mechanical properties of this hydrogel system is currently being further tested to further evaluate its suitability for fully integrated next generation prosthetic socket components.

## Conclusion

In this work a non-cytotoxic nano-clay hydrogel system was successfully developed and evaluated through rheological, mechanical and cell culture testing. Viscosity measurements revealed that the material has both thixotropic and shear thinning behaviours which makes it suitable for extrusion-based AM. The material was successfully printed using a syringe extrusion method and it was shown to have self-supporting abilities meaning that a printing-then-curing approach could be used. However, the tensile properties of the printed samples compared to the casted samples were significantly lower, likely caused by the differences in aging times. Cytotoxicity evaluations showed no significant difference in cell viability between the different conditions and cells were also found to be growing on the hydrogel surface with extended neurites present. This result indicates that the developed material shows promise for being used within biomedical applications, however, further work such as elastic modulus optimisation is required.

## Supplemental Material

supplementary_data – Supplemental material for 3D-printable zwitterionic nano-composite hydrogel system for biomedical applications Click here for additional data file. Supplemental material, supplementary_data for 3D-printable zwitterionic nano-composite hydrogel system for biomedical applications by Nathalie Sällström, Andrew Capel, Mark P Lewis, Daniel S Engstrøm and Simon Martin in Journal of Tissue Engineering
