# Supplementary material for: 3D-printable zwitterionic nano-composite hydrogel system for biomedical applications
Source: J Tissue Eng. 2020 Oct 29;11:2041731420967294. doi: 10.1177/2041731420967294 (PMC7604982; doi:10.1177/2041731420967294)
Supplement: supplementary_data – Supplemental material for 3D-printable zwitterionic nano-composite hydrogel system for biomedical applications [file supplementary_data.pdf]

Supporting information

Supplementary Table 1: Tensile data for printed samples with different pre-gel aging times. Values are reported as mean ± standard deviation (SD). This data demonstrates that there is no significant difference between tensile properties of samples aged for 5-9 h and 48 h which indicates that aging times of 24h and 48 h should be comparable.

AGING TIME	STRESS AT BREAK (kPa)	ELONGATION AT BREAK (%)	YOUNGS MODULUS (kPa)
**5-9 h**	15.0±1.1	119.8±19.3	0.489±0.180
**48 h**	16.7±2.1	91.9±15.0	0.657±0.172

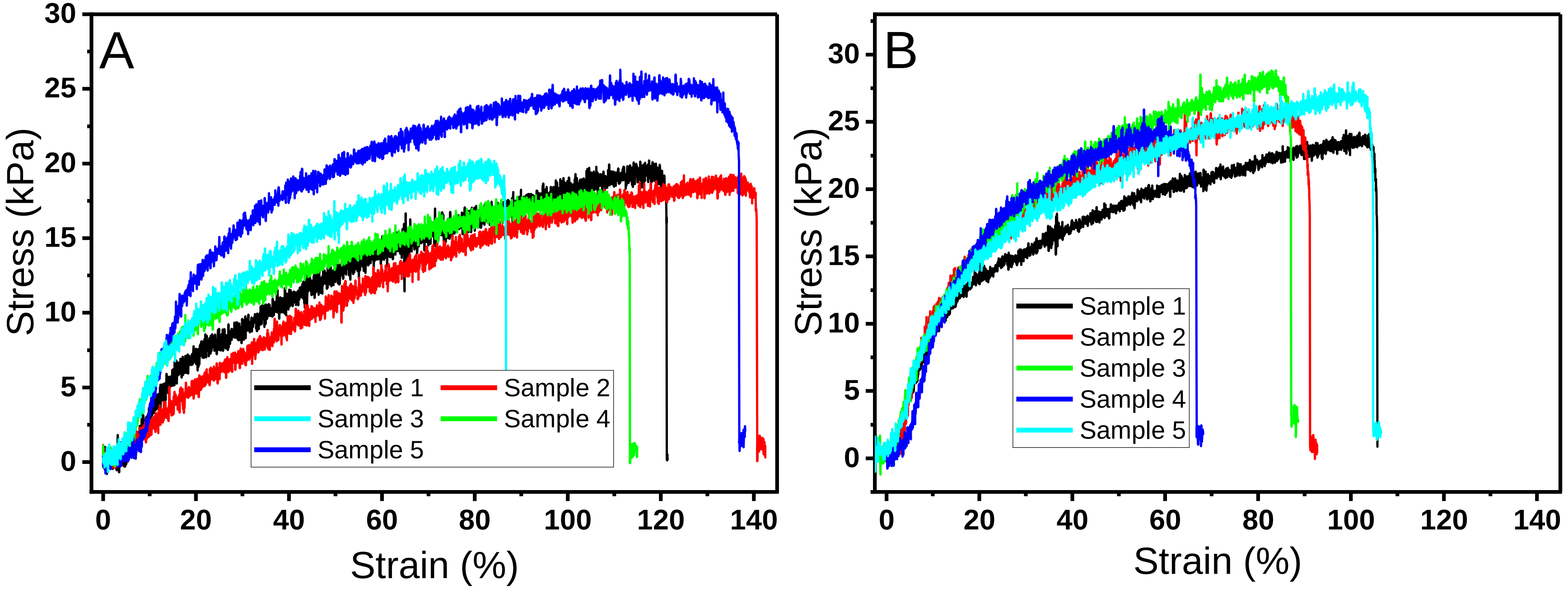

Supplementary Figure 1: Tensile stress and strain graphs for differently aged printed samples. A – Sample 1 was aged for 5 h before printing and curing and each consecutive sample was aged for one additional h meaning that sample 5 was aged for 9 h. B – all samples were aged for 48 h before printing and curing.
